# The fucosylated CD147 enhances the autophagy in epithelial ovarian cancer cells

**DOI:** 10.18632/oncotarget.13289

**Published:** 2016-11-11

**Authors:** Zhenhua Hu, Mingbo Cai, Lu Deng, Liancheng Zhu, Jian Gao, Mingzi Tan, Juanjuan Liu, Bei Lin

**Affiliations:** ^1^ Department of Obstetrics and Gynecology, Shengjing Hospital Affiliated to China Medical University, Shenyang, Liaoning, 110004, China; ^2^ Department of Obstetrics and Gynecology, The First Affiliated Hospital of Zhengzhou University, Zhengzhou, Henan, 450052, China

**Keywords:** Lewis y, CD147, autophagy, PI3K, mTOR

## Abstract

Autophagy is modulated by multiple factors including CD147, but little is know about the effects and mechanism by which the modification of CD147 by Lewis y antigen regulates autophagy of ovarian cancer cell. Here, we reported that Lewis y antigen can promote basic autophagy activity and restrain autophagic cell death in ovarian cancer cells. Furthermore, human whole genome expression profile microarrays and massage pathway analysis revealed that during early stages of autophagy in ovarian cancer cells with highly expressing Lewis y antigen, PI3K/Akt-mTOR activity was reduced, in contrast, the PI3K/Akt-mTOR signaling pathway was activated as the length of amino acid deprivation increased, which inhibited eIF4G2 expression, further decreased the transcription of autophagy-related genes, suppressed autophagic cell death. we also elaborated that co-regulates protein degradation in cells via the ubiquitin-proteasome system and the autophagy-lysosome pathway. These findings suggested that the modification of CD147 by Lewis y antigen enhanced the survival ability by promoting basic autophagy activity and restraining autophagic cell death in ovarian cancer, thus playing an important role in ovarian cancer malignant progression.

## INTRODUCTION

Due to its position, the ovary is difficult to image and monitor for abnormal growth; thus over 70% of patients with ovarian cancer are diagnosed at an advanced stage. In fact, ovarian cancer has the highest mortality rate among all gynecological malignancies. It has been previously demonstrated that during the growth of malignant tumor cells, rapid proliferation can result in serious hypoxia and nutrient deficiencies in local tissues. Tumor cells can adapt to such harsh environmental changes by regulating their own metabolism, changing DNA repair, or inducing angiogenesis among other mechanisms [8, 31]. Ovarian cancer develops rapidly and adverse environmental changes including hypoxia, nutrition shortage, and inflammatory conditions predominate as the tumor becomes malignant. Therefore, it is essential to explore the mechanisms by which ovarian cancer cells adapt to hypoxia and starvation in order to better control tumor progression.

Autophagy is a process by which cells degrade their own damaged organelles and macromolecular substances by utilizing lysosomes. Autophagy is unique to eukaryotic cells, in both normal and pathological tissues. Current studies have indicated that autophagy is both helpful and harmful to the tumor development process. When cancer cells lack energy, autophagy is activated and internal substances are then degraded to provide energy and promote cell survival. However, excessive autophagy can also lead to autophagic cell death, known as type II programmed cell death [13]. The triggering, progression and outcome of autophagy are precisely regulated in which several proteins and signaling pathways are involved, however, the exact regulation mechanism remains unclear.

CD147, a glycoprotein and a member of the immunoglobulin superfamily, is highly expressed in several malignant tumors. Expressed at high level, CD147 can promote the invasion and metastasis of tumor cells [35, 39], inhibit cell apoptosis and anoikis [21, 23], contribute to neo-vascularization and enhance resistance to several chemotherapeutic drugs [3, 17]. Gou X et al. reported that CD147 involvement in the development of liver cancer is related to restrain autophagic cell death in liver cancer [12]. Our preliminary studies have shown that CD147 is highly expressed in ovarian cancer tissues and promotes the resistance of ovarian cancer cells [42]. As different sugar chains are added, the CD147 protein is present in both low (LG-CD147, ~36kDa) and highly glycosylated (HG-CD147, ~40–60 kDa) forms. The level of glycosylation influences the function of the CD147 protein; differently glycosylated forms of CD147 have been shown to have varying functions. Jia L et al. reported that the highly glycosylated CD147 molecule plays a key role in the invasion and metastasis of tumor cells, while a purified de-glycosylated CD147 molecule lacked the ability to induce matrix metalloproteinases and exhibited decreased *in vitro* adhesion capability [18].

Lewis y antigen, a tumor-related carbohydrate antigen, is an oligosaccharide chain containing a bi-fucosyl group. It is an important component of many glycoproteins and glycolipids on the cell surface and it functions to receive the transmission of several intracellular and extracellular signals as a cell surface “antenna”. In a preliminary study, our research group investigated the relationship between Lewis y antigen and the occurrence and development of ovarian cancer. We found that the ovarian cancer cell lines with high levels of Lewis y antigen expression showed accelerated proliferation, reduced apoptosis, shortened cell cycle, and enhanced *in vivo* oncogenicity; after blockage with a monoclonal antibody against Lewis y antigen, the malignant behaviors of the cells were significantly weakened [11, 25, 40]. Furthermore, our preliminary work also indicated that Lewis y antigen is a part of the CD147 protein structure and that increased expression of Lewis y antigen strengthened the ability of CD147 to promote the adhesion and invasion of ovarian cancer cells [10].

Autophagy is controlled by a series of signaling pathways. Current studies have suggested that Class I PI3K is a negative regulator of autophagy, while Class III PI3K can phosphorylate phosphatidylinositols (PtdIns) to produce 3-phosphatidylinositol phosphate and promote the occurrence of autophagy [7, 26]. Our preliminary results have shown that Lewis y antigen over-expression promotes the proliferation of ovarian cancer cells via the Class I PI3K/Akt signaling pathway [25].

Proteins within the cell are degraded mainly via two pathways: autophagy and the ubiquitin-proteasome system (UPS). Recent studies have revealed that UPS and autophagy-lysosome system are closely related and are co-regulated. It has been found that the lack of proteasome function can activate autophagy and autophagy activation can offset the loss of proteasome function [28]. In addition, eliminating autophagy can suppress proteasome function and cause the accumulation of poly-ubiquitinated proteins [34].

Thus, this study has the following objectives: (1) to determine the role of CD147 in autophagy and autophagic death of ovarian cancer cells; (2) to clarify whether a fucosylated Lewis y antigen on the CD147 molecule affects the ability of CD147 to regulate autophagy in ovarian cancer cells; (3) to explore the mechanism by which Lewis y antigen can regulate CD147 and thus the autophagy of ovarian cancer cells; and (4) to analyze whether the involvement of Lewis y antigen in regulating the autophagy of ovarian cancer cells is related to the UPS.

## RESULTS

### CD147 expression in the ovarian cancer cell autophagy model

**Figure 1:**
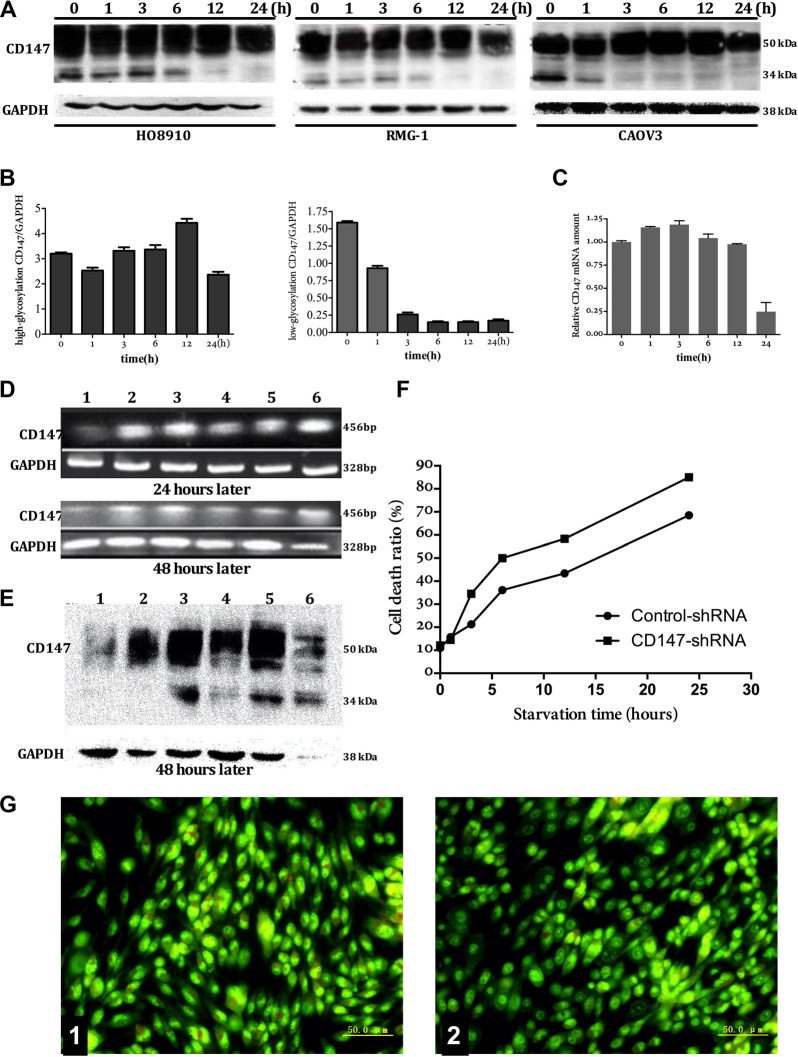
The relationship between expression of CD147 and autophagy The expression of CD147 protein in three types of ovarian cancer cell (HO8910, RMG-1, CAOV3) on mRNA and protein level (Figure **A**, **B**, and **C**): mRNA remained stable at a high level as the time extend after amino acid deprivation. the LG-CD147 protein was significantly decreased in HO8910 and RMG-1 cells at 6 h and completely undetectable by 12 h after amino acid deprivation. In contrast, LG-CD147 was reduced at 1 h and then undetectable by 3 h after amino acid deprivation in CAOV3 cells, then, HG-CD147 protein expression remained stable at a high level as amino acid deprivation time prolong. In order to further clarify the relationship between the continuous high expression of CD147 and the autophagic death in tumor cells, we reduced CD147 expression using shRNA, we found that oligo-nucleotide fragments BSG-1211 began to interfere CD147 gene transcription after interference for 24 hours, and obviously interfered CD147 gene transcription after interference for 48 hours (1 = BSG-1211; 2 = BSG-853; 3 = BSG-941; 5 = BSG-1024; 5 = Negative control; 6 = Positive control). the expression of CD147 on protein level after shRNA-CD147 interference for 48 hours, indicating that the protein level of CD147 correspondence with the mRNA levels (Figure **D**, **E**). the rate of cell mortality after shRNA-CD147 interference, there was significant differences between shRNA-CD147 interference group and control group (without interference) after amino acid deprivation for 6 hours, 12 hours, 24 hours (Figure **F**). The variation of intracellular autophagic vacuole number by AO staining before or after shRNA-CD147 interference, the number of intracellular autophagic vacuole obviously reduced after sh-CD147 interference (Figure G, 1: control; 2: sh-CD147 interference).

At 1 h, 3 h, 6 h and 12 h after amino acid deprivation, CD147 mRNA and protein expression remained stable at a high level in three types of ovarian cancer cell lines tested; however, CD147 levels decreased at 24 h. In each of the three cell lines, LG-CD147 protein expression disappeared at different time points after amino acid deprivation. For example, the LG-CD147 protein was significantly decreased in HO8910 and RMG-1 cells at 6 h and completely undetectable by 12h after amino acid deprivation. In contrast, LG-CD147 was reduced at 1h and then undetectable by 3 h after amino acid deprivation in CAOV3 cells, then, HG-CD147 expression stable at a high level in three types of ovarian cancer cell (Figure 1A–1C).

### CD147 expression and the variation of cell autophagic death after shRNA-CD147 interference

In order to further clarify the relationship between the continuous high expression of CD147 and the autophagic death in tumor cells, we reduced CD147 expression using shRNA. We found that the shRNA, BSG-1211, could significantly suppress the transcription and translation of the CD147/*BSG* gene at 24 h after transfection (Figure 1D, 1E). After shRNA-mediated downregulation of CD147 expression, changesof autophagic vacuole and cell morphology and rate of cell death within two group cells were observed using AO stain and trypan blue experiment, the results indicated that, compared with the control shRNA group, autophagic vacuole reduced markedly and the cell death rate in CD147-shRNA transfected cells was significantly increased at 6 h, 12 h and 24 h after amino acid deprivation (Figure 1F, 1G).

### Expression of FUT1, Lewis y antigen, CD147, and Lewis y antigen coupled to CD147 in ovarian cancer cells before and after FUT1 transfection

**Figure 2:**
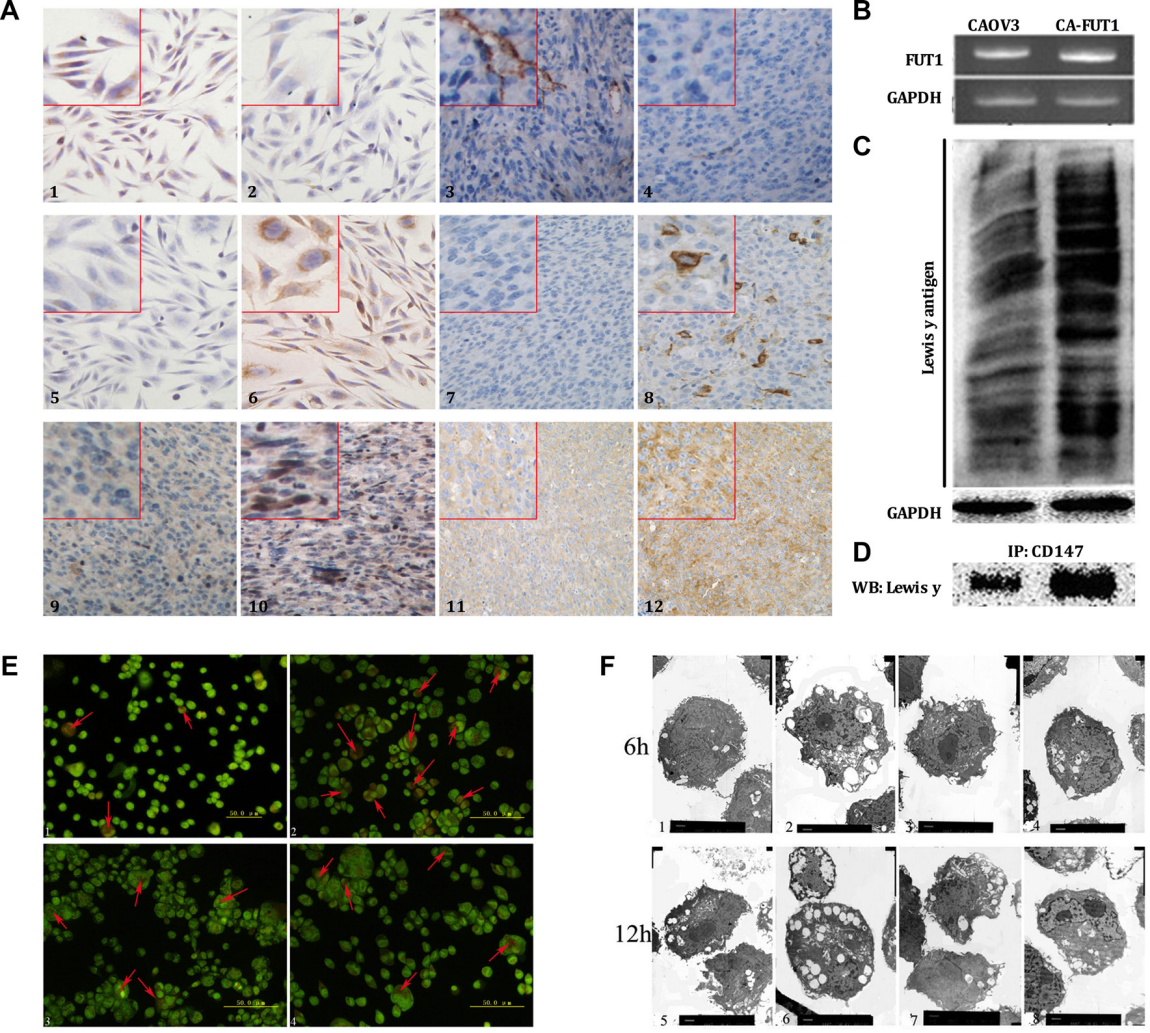
The effects of Lewis y antigen on CD147 regulated autophagy of ovarian cancer cells The detection of Lewis x, Lewis y antigen, CD147 and Beclin 1 protein expression by immunocytochemistry and immunohistochemistry (Figure **A**, 1~4 is Lewis X antigen, 5~8 is Lewis Y antigen, 9~10 is CD147molecule, 11~12 is Beclin 1 protein. 1, 3, 5, 7, 9 and 11 is CAOV3, cells before transfection; 2, 4, 6, 8, 10 and 12 is CAOV3 after transfection, CA-FUT1 cells), we can see that the expression of Lewis y antigen, Beclin 1 and CD147 increased after transfection of FUT1. The expression of *FUT1* gene before and after transfected *FUT1* gene tested by PCR (Figure **B**). The expression of whole Lewis y antigen before and after transfection tested by western-blot (Figure **C**). The Lewis y antigen on CD147 molecule before and after transfection tested by immunoprecipitation (Figure **D**). In order to investigate the role of Lewis y antigen in promoting autophagy, we measured changes in autophagic flux in cells using AO staining and transmission electron microscopy: the numbers of intracellular autophagic vacuole tested by AO staining; the number of autophagic vacuole and cellular morphology observed by transmission electron microscope (Figure **E** and **F**, e1 f1and f5 is CAOV3, cells before transfection; e2 f2 and f6 is CA-FUT1, cells after transfection; e3 f3 and f7 is CA-FUT1 cells treated by Lewis y monoclonal antibody; e4 f4 and f8 is CA-FUT1 cells treated by 3-MA).

FUT1 is a key enzyme in the synthesis of the Lewis y antigen. Lewis y antigen is synthesized through FUT1 regulation and the addition of a fucose molecule at the terminal of Lewis x antigen under the catalysis of FUT1. In order to further investigate the effects of Lewis y antigen on CD147-regulated autophagy of ovarian cancer cells, we transfected CAOV3 cells with FUT1; the resulting cells are referred to as CAOV3-FUT1 cells (CA-FUT1). The expression of Lewis x and Lewis y antigens before and after transfection were determined using immunocytochemistry. CAOV3 cells, which express Lewis x antigen yet very low levels of Lewis y antigen, significantly increased expression of Lewis y antigen after transfection of FUT1. In addition, we established a nude mouse transplantation tumor model, transplanting untransfected cells as well as FUT1-transfected cells and then determined changes in expression of the Lewis x and Lewis y antigens using immunohistochemistry. The results were in agreement with the data from the *in vitro* model. Meanwhile, in the nude mouse transplantation model, CD147 and Beclin 1 protein was also increased in tumor cells transfected with FUT1 as compared to untransfected cells (Figure 2A). The mRNA level of FUT1gene was markedly increased in the transfected cells (Figure 2B). Changes in total Lewis y antigen expression within-cells were detected by Western blot (Figure 2C); the data demonstrated that the expression of Lewis y antigen in CAOV3-FUT1 cells was increased to different degrees. We also found that the amount of Lewis y antigen on the CD147 molecules increased after transfection using immunoprecipitation (Figure 2D).

### Changes of cell autophagy before and after FUT1 transfection

In order to investigate the role of Lewis y antigen in promoting autophagy, we divided cells into four groups (CAOV3, CA-FUT1, Lewis y antibody add CA-FUT1, 3-MA add CA-FUT1) and then measured changes in autophagic vacuole in cells using acridine orange (AO) staining. We found that the number of acidic vesicles was increased in the cells that highly expressed Lewis y antigen at 6 h after amino acid deprivation, but lower numbers of acidic vesicles after Lewis y antibody block CA-FUT1 cell A widely used autophagic inhibitor, 3-methyladenine (3-MA), can inhibit the formation of the Class III PI3K/Beclin1 complex and specifically block the fusion of autophagosomes and lysosomes during autophagy. We found a significant decrease in the number of acidic vesicles at 6 h after CA-FUT1 treatment with 3-MA (Figure 2E). We then used transmission electron microscopy (TEM) to determine the number and morphology of autophagic vesicles in cells (Figure 2F). We found that the number of autophagic vesicles was increased in cells with high levels of Lewis y antigen expression at 6 h after amino acid starvation. At 12 h after amino acid deprivation, the number of acidic vesicles was increased in the transfected group; yet, untransfected cells and cells treated with 3-MA to block autophagolysosome formation exhibited more broken cell. Thus, by blocking autophagosome and lysosome fusion, 3-MA interrupted the circulation of energy and macromolecular substances resulting in the broken of numerous cells. These results suggest that Lewis y antigen can promote a basal level of autophagic activity in ovarian cancer cells as well as prevent excessive autophagy and subsequent death as a result of amino acid deprivation.

### Relation between Lewis y antigen and autophagic death of ovarian cells

To further investigate the possible involvement of Lewis y antigen in cell autophagic death after amino acid deprivation, we starved CAOV3 and CA-FUT1 cells for 6 h or 12 h and then trypan blue experiment. After 12 h starvation, there was a significant difference in the cell death rate between the CA-FUT1 cell group and CAOV3cell group; in addition, after 3-MA treatment, CA-FUT1 transfected cells had a lower death rate than CAOV3 cells (Table 1).

**Table 1:** The ratio of cell death among groups

	Cell death ratio (%)	
Groups	6 h	12 h
CAOV3	34.0	67.1
CA-FUT1	35.7	56.5
CA-FUT1+LeY Antibody	36.3	65.5
CA-FUT1+3-MA	36.1	52.0

### *BECN1*/Beclin1 (Atg6) expression before and after FUT1 transfection

**Figure 3:**
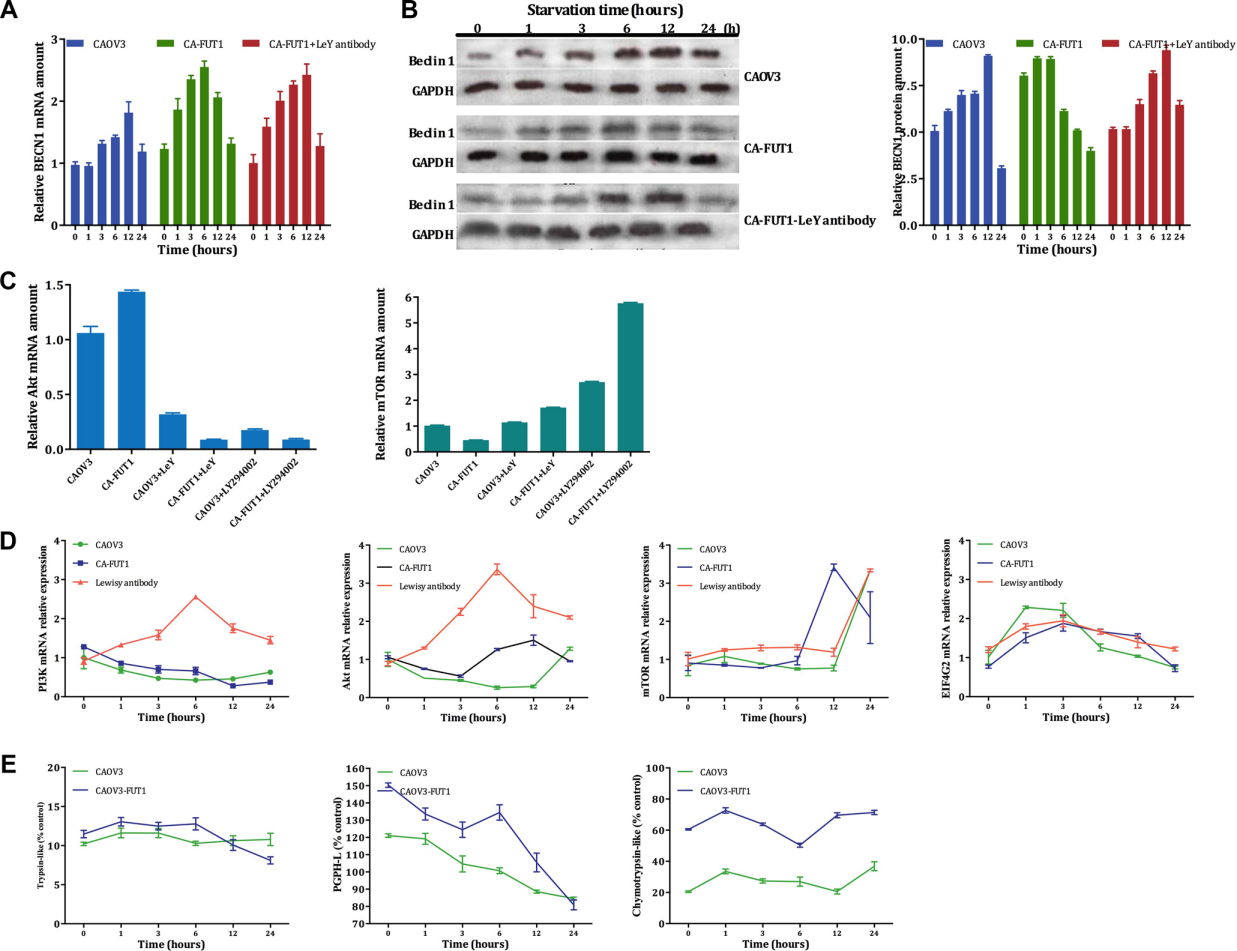
The expression of *BECN1* gene tested by Real-Time PCR after starvation three group cells (Figure A) The Beclin 1 protein tested by Western-blot in three model of autophagy (Figure **B**, **C**). and Real-Time PCR to validate Akt and mTOR molecular, the results found via gene chip (Figure **D**, **E**).

*BECN1* (an autophagy-related factor) mRNA expression changes were detected using real-time PCR. At the early stage of autophagy (0 h, 1 h, 3 h, 6 h after amino acid deprivation), BECN1 mRNA expression was gradually increased in CA-FUT1 cells until it reached a peak at 6 h after amino acid-deprivation, levels were higher than in CAOV3 cells. Lewis y antibody inhibiting experiment indicating that Lewis y antibody could partially block this effect (Figure 3A). Using immunoblot analysis, Beclin1 protein expression changed along with changes in its mRNA expression. During autophagy, the ratio of *Beclin1/Gapdh* in CA-FUT1 cells progressively increased until a peak at 6h but then significantly decreased at 12 h after amino acid deprivation. Lewis y antibody blockage could partially reverse Beclin1 protein expression. In CAOV3 cells, Beclin1 protein expression also increased with time of amino acid deprivation to a peak at 12 h then significantly decreased at 24 h (Figure 3B). These findings suggested that the basal level of autophagy in FUT1-transfected cells was increased.

### Transcriptional changes in key molecules in the PI3K/Akt-mTOR-EIF4G signaling pathway after FUT1 transfection

To further study the mechanism by which Lewis y antigen regulates basal levels of autophagy and inhibits autophagic cell death, we conducted whole genome gene expression chip screening comparing untransfected and FUT1-transfected cells, analyzing transcriptional changes in genes involved in autophagy-related signaling pathways. Our results indicate that three signaling molecules (mTOR, PTEN, and eIF4G_2_) in the mTOR signaling pathway were decreased in FUT1-transfected cells. We also found that PI3K mRNA had no significant changes, but Akt mRNA was increased. Subsequently, we treated untransfected and FUT1-transfected with a monoclonal antibody (Lewis y antibody) to the Lewis y antigen or with LY294002 (a PI3K inhibitor) then measured mTOR, and AKT mRNA by RT-PCR to validate the results found via gene chip. Again, we found after Lewis y antibody blockage or LY294002 treatment, Akt mRNA levels increased and mTOR mRNA levels decreased (Figure 3C). These data suggest that the PI3K-Akt-mTOR-eIF4G2 signaling pathway plays a regulatory role in the autophagic process by which Lewis y antigen enhances basal autophagy and protects ovarian cancer cells from autophagic cell death.

### PI3K/Akt-mTOR signaling pathway activity after transfection with FUT1

CAOV3 and CA-FUT1 and anti-Lewis y antigen monoclonal antibody treated CA-FUT1 Cells, next, mRNA levels of PI3K, Akt, and mTOR were measured using real-time PCR. In untransfected cells, PI3K and Akt mRNA levels decreased at the early times after amino acid starvation (1 h, 3 h, and 6 h), but then increased after longer times of starvation (12 h and 24 h). Similarly, after FUT1 transfection, PI3K and Akt mRNA levels increased than CAOV3 cells under conditions of complete nutrition, yet also decreased after amino acid deprivation. This effect could be partially reversed using anti-Lewis y antibody blockage. At the early stages of nutrition deprivation for all cells, mTOR mRNA expression exhibited no significant changes but then increased at later times after-amino acid deprivation. After FUT1 transfection, mTOR expression suddenly increased at 12 h of starvation; while without transfection and antibody blockage, mTOR expression increased at 24 h of starvation. eIF4G2, an downstream target of mTOR, increased in both untransfected and FUT1 transfected cells at early stages of starvation; yet, eIF4G2 was down-regulated in the transfected cells at ≥ 6 h of starvation and in the untransfected cells at ≥ 24 h of starvation (Figure 3D). These findings suggest that at the early stages of starvation, basal autophagy in transfected cells is increased, PI3K/Akt-mTOR activity is decreased, and the expression of eIF4G2 (an mTOR downstream target and an mRNA transcription regulatory factor) is enhanced, which promotes the transcription of autophagy-related genes. As the starvation lengthens, the PI3K/Akt-mTOR signaling pathway is activated under the continuous stress of both the internal and external metabolism environments. As eIF4G2 expression is suppressed, transcription autophagy-related genes decreases, and excessive autophagy of cells is inhibited. These results reveal that highly expressed Lewis y antigen participates in the regulation of cellular autophagy activity and increases the self-contained energy supply in cells resulting in enhanced survival in malignant environments.

### UPS activity assay

The ubiquitin proteasome system (UPS) is closely related to the autophagy-lysosome system and they mutually regulate protein degradation in cells. Basal levels of autophagy in transfected cells are increased and there is “autophagy flux” accumulation in cells during early stages of amino acid deprivation; in other words, degradation of proteins and/or broken organelles is increased. How about the correlations of the accumulation of these substances to be degraded and the increased expression of CD147 and other proteins in the transfected cells with UPS activity? In order to further determine the relationship between autophagosomes, inhibited proteasome activity, and increased expression of CD147 and other proteins in FUT1-transfected cells, we established a model of autophagy using CAOV3and CA-FUT1cells and then measured the activity of three different proteasomes. We found that trypsin-like proteasome activity was not significantly changed in either CAOV3or CA-FUT1cells during early stages of autophagy, but then decreased in transfected cells at ≥ 12 h after amino acid deprivation. Peptidyl glutamyl-peptide hydrolase-like proteasome activity was increased in CA-FUT1cells during the early stages of autophagy, then decreased over time. By contrast, chymotrypsin-like proteasome activity was increased in CA-FUT1cells and remained at a high level (Figure 3E). These results suggest that autophagy and proteasome activity in FUT1-transfected ovarian cancer cells initial increases during amino acid deprivation, while by 12 h after amino acid deprivation, autophagic activity peaks and autophagic cell death begins to occur. Then proteasome activity decreases to prevent further autophagic cell death. Thus, there is a close relationship between the autophagy-lysosome system and the UPS, which act to regulate protein degradation in cells cooperatively.

## DISCUSSION

Autophagy is a behavior that helps cells to survive in nutrient lack or other stress conditions by inducing digestion of their own proteins, organelles, and cytoplasm. There are two types of cell autophagy: (1) autophagy resulting from extracellular conditions, such as nutrition deprivation in the external environment, ischemia-hypoxia, or decreased concentrations of growth factors and (2) autophagy resulting from intracellular conditions such as metabolic pressure, aging, broken-organelles, or incorrectly folded or aggregated proteins. These two types of cellular autophagy can be induced separately but may also happen concurrently [29]. One study has suggested that starvation is the most important inducer of autophagy in eukaryotes [6].

As a member of the immunoglobulin superfamily, CD147 is highly expressed in several malignant tumor cell types including ovarian cancer cells. It mainly participates in cell-cell or cell-matrix interactions, stimulating adjacent fibroblasts to secrete a large numbers of matrix metalloproteinases (MMPs) and affects the invasion and metastasis of tumors. In addition, CD147 can suppress apoptosis and anoikis [21, 38], promote neovascularization, enhance resistance to several chemotherapeutic drugs [3, 17], promote the use of glycolysis in energy metabolism, and protect tumor cells from death [14, 20]. Another study has shown that CD147 is associated with autophagy [12], but did not identify a mechanism.

In the present study, we established several autophagy models in ovarian cancer cells utilizing total amino acid deprivation to determine the role of CD147 in autophagy and autophagic cell death. Our results indicate that, under the continuous stress in both the extracellular and intracellular environment, CD147 mRNA transcription was maintained at a high level, yet was significantly decreased after 24 h of amino acid deprivation. Total CD147 protein levels were consistent with CD147 mRNA transcription. In CAOV3 cells, shRNA was used to decrease CD147 mRNA and protein expression. An autophagy model was established by interfering with CD147 expression; the death rate in the CD147-shRNA interference group was significantly increased at 6 h, 12 h, and 24 h after amino acid deprivation. These findings suggest that the high expression of CD147 protein participates in the autophagy process of ovarian cancer cells and can prevent autophagic cell death.

In three cell autophagy models, there was a significant change in the glycosylation state of CD147. HG- CD147 protein expression either increased or remained highly expressed after short times of amino acid deprivation. While LG-CD147 protein expression was decreased after amino acid deprivation until it became completely undetectable. These data indicate that HG-CD147 plays a major role in autophagy and protecting cells from autophagic cell death. A recent study reports that CD147 is a highly glycosylated transmembrane glycoprotein, its outer-membrane domain contains three N-binding glycosylation sites (N44Q, N152Q and N186Q), and its molecular weight fluctuates between 31–65 kDa due to diverse levels of N-terminal glycosylation [2, 9]. CD147 is present in two forms: lowly glycosylated CD147 (LG-CD147) and highly glycosylated CD147 (HG-CD147). LG-CD147 is rich in mannose and it exists in the cytoplasm. In contrast, HG- CD147 is a transmembrane protein whose main sugar chain is a β 1,6-polylactosamine chain; in addition, several forms of glycosylation such as galactosylation, fucosylation, saliva acidification occur on the lateral branches of the main sugar chain. The synthesis of N-binding sugar chains begins in the endoplasmic reticulum and finished in the Golgi. Glycoproteins produced in the endoplasmic reticulum have similar sugar chains. After the cis surface enters the Golgi body, a series of ordered processing and modification happen that facilitate the process of transportation among various membrane vesicles. Most mannose in the original sugar chain is removed in the Golgi and different types of sugar molecules are added in sequence by corresponding glycosyltransferases to form oligosaccharide chains with different structures. The spatial structure of glycoproteins determines the glycosyltransferases which bind them, resulting in specific glycosylation modifications [4]. The degree of glycosylation degree determines the molecular weight of a molecule. Glycoproteins with higher levels of glycosylation tend to have stronger activity, including greater self-polymerization capability and ligand-binding affinity [41]. Several investigators have shown that glycosylation degree determines the activity of the ability of the CD147 molecule to stimulate fibroblasts and tumor cells to secrete MMPs; highly glycosylated transmembrane CD147 interacts with the monocarboxylate transporter (MCT), cyclosporin A, and integrin to promote cellular adhesion, mediate lactic acid transport, and induce signal conversion in cells [18, 33].

Purified, deglycosylated CD147 (LG-CD147) does not induce matrix metalloproteinases, has no self-polymerization capability, and it has reduced *in vitro* adhesion capability [18]This study has proven that the HG-CD147 molecule participates in the adaption of ovarian cancer cells to their adverse environment, prevents cells from autophagic cell death, At the early stages of cell starvation, intracellular protein synthesis function is reduced, LG-CD147 synthesis is decreased, and existing LG-CD147 is converted to HG- CD147 to guarantee the stability of HG-CD147 protein expression. Gao et al. suggests that high expression of CD147 protein can prevent liver cancer cells from autophagic cell death by down-regulating ATG6/Beclin1 expression [12], which is consistent with our study results. In this study, we are the first to report that HG-CD147 protein plays a leading role in preventing ovarian cancer cells from excessive autophagy and autophagic cell death.

Autophagic death has different biological characteristics than apoptosis, specifically there are a large number of vacuole structures enclosing the cytoplasm and the contents of the vacuoles are degraded by fusion with lysosomes; during this process, the mitochondria are fully functional and provide energy for autophagy. The TEM observations in this study showed that before transfection and after Lewis y antibody blockage, a large number of cells were shrunken, broken and no longer viable by 12 h after amino acid deprivation; after FUT1-transfection, the number of autophagic vacuoles was significantly increased at 6 h after starvation and the number of acidic vesicles increased by 12 h after amino acid deprivation, with some cell death was evident. This was comparable to the cell death rate after treatment with 3-MA (an autophagy inhibitor). These data indicate that 12 h amino acid deprivation induces strong autophagy activity in ovarian cancer cells and that highly expressed Lewis y antigen can inhibit cell death including autophagic cell death under harsh internal and external conditions by promoting the adaption of cell autophagy to the abnormal environment. Another study showed that nutrient deprivation induced intense autophagy and caused the atrophy of partial cancer cells as well their entry into a reversible resting state. These resting cells might be able to exit from this state and re-enter the cell cycle if the microenvironment changed, for example, if more nutrition was obtained [30]. This study demonstrated that, in nude mice transplanted with tumor cells, CD147 expression was generally increased and Lewis y antigen was focally and highly expressed in the central part of the resulting tumor. Therefore, we propose that increased expression of Lewis y antigen plays a major role in preventing autophagic cell death and that high expression of Lewis y antigen can promote the entry of cells into a reversible resting state required for survival in an adverse environment.

The mTOR kinase senses changes in amino acids, ATP levels, and hormones; it regulates autophagy and one of its downstream targets is 4E-binding protein 1 (4E-BP1, an initiation factor regulating eukaryotic protein synthesis) which is an important inhibitor of eIF4E [5]. mRNA translation initiation is the binding of ribosomes with mature mRNA, which requires the cooperation of numerous eukaryotic translation initiation factors (eIFs) such as eIF4E and eIF4G. eIF4G plays a “scaffold” role [32] ; eIF4G2 is a subunit of eIF4G essential for protein translation in eukaryotic cells. In order to further explore the mechanism by which Lewis y antigen enhances CD147-mediated autophagy, we conducted whole genome chip screening with untransfected and FUT1-transfected cells and analyzed changes in autophagy-related signaling pathways. The results indicated that there was a significant change in the transcription signaling molecules in the PI3K/Akt-mTOR-eIF4G2 signaling pathway. We validated the chip results using fluorescence-labeled real-time PCR; our results showed that at the early stages of autophagy (1–6 h of starvation), the transcription activities of signaling molecules in the PI3K/Akt-mTOR-eIF4G2 signaling pathway were lower in FUT1-transfected cells: PI3K/Akt transcription was decreased and the expression of eIF4G2 (a downstream signaling molecule of the mTOR protein) was increased. These results suggest that high expression of eIF4G2 (an mRNA transcription initiation regulator) promotes the transcription of autophagy-related genes, so as to ensure that high energy requirements of tumor cells at the early stage of external nutrition deprivation are met. After 6h starvation, when intense autophagy was induced by extracellular starvation and intracellular metabolism stress, PI3K/Akt is activated, which inhibits transcription of eIF4G, Beclin1 and other autophagy regulating proteins resulting in reduced autophagic cell death. Thus, these findings indicate that the PI3K/Akt-mTOR- eIF4G2 signaling pathway is a sensitive sensor of metabolic stresses in both the extracellular and intracellular environments and Lewis y antigen participates in regulating autophagy-autophagic cell death in ovarian cancer cells likely via this signaling pathway.

Autophagy and the UPS pathway are two of the most important pathways involved in protein degradation within cells; current studies have shown that they are closely related to each other [1]. In order to further investigate whether the increased expression of Lewis y antigen enhanced the expression and activity of several cell surface receptors including CD147 and whether the increased autophagic activity at the early stages was associated with changes in proteasome activity, we measured the activity three types of proteasome activity in untransfected and FUT1-transfected cells. Our results revealed that the activities of these three proteasomes were increased in ovarian cancer cells of high-grade malignancy which allowed the cells to maintain a higher metabolic rate. Kondakova IV et al. reported that, compared with normal tissues, chymotrypsin activity was significantly increased in ovarian cancer tissues and much higher in peritoneal metastatic tissue [22]. This result is consistent with the conclusions in our study; both results are also consistent with the latest studies on the use of proteasome inhibitors in the targeted tumor therapy. Iunusova et al. reported that 26S-proteasome and calpain activity were both decreased in tissue specimens from ovarian cancer patients with ascites, making it difficult to form definitive conclusions regarding the role of proteasomes in the malignant progression of ovarian cancer [15]. However, the data do demonstrate that at the early stages of autophagy, the activity of the peptidyl glutamyl-peptide hydrolase-like and chymotrypsin-like proteasomes were increased in ovarian cancer cells of high-grade malignancy, but when excessive autophagy of cells was induced by external and internal harsh environments autophagic death occurred in a large number of cells and the activities of trypsin-like and peptidyl glutamyl-peptide hydrolase-like proteasomes were decreased. These data indicate that the UPS and autophagy-lysosome system play cooperative roles in degrading the damaged proteins so as to maintain a dynamic equilibrium in tumor cells. Kao et al. reported bortezomib, a proteasome inhibitor, hindered the formation of autophagic flux by suppressing the activity of tissue lipase in lysosomes [19]; our preliminary studies found that proteasome inhibitors induced Beclin 1-independent autophagy in ovarian cancer cells and the later strengthened the toxic effects of the former on ovarian cancer cells in turn [24]. The studies described here suggest that proteasome system is closely linked to the autophagy-lysosome system and that they play either a cooperative or antagonistic role depending on the environment.

Our data demonstrated that the modification of CD147 by Lewis y antigen enhanced the survival ability by promoting basic autophagy activity and restraining autophagic cell death in ovarian cancer cell, thus playing an important role in ovarian cancer malignant progression.

## MATERIALS AND METHODS

### Materials

RMG-I cells are derived from human ovarian clear cell carcinoma tissues and were given from Professor Iwamori Masao in the University of Tokyo [16]. CAOV3 and HO8910 cells are derived from ovarian serous cell carcinoma and were generated by our research group(NTCC, Biovector Science Lab,Inc). The fluorogenic peptide substrates (Suc-Leu-Leu-Val-Tyr-AMC, chymotrypsin-like; Z-Val-Val-Arg-AMC, trypsin-like; Z-Leu-Leu-Glu-AMC, peptidyl glutamyl-peptide hydrolase-like) were synthesized by Sangon Biotech Co., Ltd (Shanghai, China).

### Establishment of a cell autophagy model

Cells were inoculated onto a 6-well plate at 6 × 10^5^ cells/well and then incubated 24 h at 37 and 5% CO_2_. Thereafter, these cells were washed twice with Earle's balanced salt solution (EBSS) and then given a fresh 2 ml EBSS. then, the cells were cultured 0 h, 1 h, 3 h, 6 h, 12 h, or 24 h in a 37, 5% CO_2_ incubator and then digested and collected for use.

### shRNA interference experiment

shRNA expression vectors targeting the CD147 gene along with random nonsense scrambled control sequences were designed and synthesized (Shanghai Genechem Co., Ltd). Lipofectamine TM 2000 Reagent (Invitrogen, Life Technologies, USA) was used for transfection of the vectors according to the manufacturer's instructions.

### Trypan blue experiment

A 10^6^/ml single cell suspension was prepared at each time point and then 100ul cell suspension was added to 100 ul 0.8% trypan blue solution and mixed evenly. Both live and dead cell counts were determined; the cell death rate = sum of dead cells / total number of cells. Each time-point was counted in triplicate.

### Establishment of cell line stably transfected by the α1, 2-FT gene, FUT1

CAOV3 cell, transfect α1, 2-FT gene(expression vector pcDNA3.1(−), pcDNA3.1-hFUT and the vector alone were transfected into CAOV3), Transfection was performed using Lipofectamine TM 2000 Reagent (Invitrogen, Life Technologies, USA) according to the manufacturer's instructions. Transfected cells were screened and selected using G418 to establish CAOV3-FUT1 and CAOV3-pcDNA3.1, respectively. The expression of FUT1 and the total Lewis y antigen within the cells was validated at the mRNA and protein levels, respectively. And Lewis y antigen on CD147 protein was validated by immunoprecipitation. Cells were collected both before and after transfection for subsequent experiments.

### Acridine orange (AO) staining and transmission electron microscopic (TEM) observation

Autophagic flux and morphology in cells were observed and detected before and after transfection, after Lewis y antibody blockage, and after 3-MA interference [37]. The concentration of AO solution was 0.1 mg/ml, and was done as previous investigations [27].

### Immunoprecipitation

Ice-cold RIPA buffer was added to the ovarian cancer cells (CAOV3, CAOV3-FUT1), followed by incubation for 30 min at 4. After centrifugation at 15000 × g for 30 min at 4, the supernatant was collected and treated with 2 ug of rabbit anti-CD147 polyclonal (proteintech, USA) for 3 h at 4. Then, 20 ul of protein A/G plus-Agarose(Santa Cruz Biotechnology, Inc) was added, followed by incubation on a rocker platform overnight at 4, the primary antibody was replaced by rabbit Ig G (Bioss, China) as negative control. Immunoprecipitates were subsequently subjected to 10% SDS gel electrohporesis and analyzed via western blot using mouse anti-Lewis y monoclonal antibody, proteins were visualized using ECL reagent (Thermo scientific ECL, USA).

### Proteasome activity assay

In order to extract proteasomes from CAOV3 and CAOV3-FUT1, the cells were thoroughly scraped from the culture dishes with a cell scraper at different time points after amino acid deprivation, and washed with cold PBS. Proteasomes were extracted according to previous description [36]. Peptidase activities of the proteasome were determined by measuring hydrolysis of the fluorogenic substrates, Suc-Leu-Leu-Val-Tyr-AMC, Z-Val-Val-Arg-AMC, and Z-Leu-Leu-Glu-AMC. These substrates are preferentially hydrolyzed by the chymotrypsin-like, the trypsin-like and the peptidyl glutamyl peptide hydrolase (PGPH)-like activities of the proteasome, respectively. Ten microliters (1 μg/μL) of each freshly made supernatant was incubated in a 96-well plate at 37°C for 30 min with 10 μL of 250 μmol/L Suc-Leu-Leu-Val-Tyr-AMC, 500 μmol/L Z-Val-Val-Arg-AMC, 500 μmol/L Z-Leu-Leu-Glu-AMC and 85 μL assay buffer (20 mmol/L Tris-HCl, pH 7.5, and 10% glycerol). DMSO (10 μL) instead of fluorogenic substrate was incubated with the proteasome supernatant and was used as the negative control. The release of fluorescent AMC was measured with a spectrofluorometer (Perkin-Elmer Life and Analytical Sciences, Inc., Wellesley, MA, USA) at 460 nm with an excitation wavelength of 380 nm.
